# Blood transcriptomic discrimination of bacterial and viral infections in the emergency department: a multi-cohort observational validation study

**DOI:** 10.1186/s12916-020-01653-3

**Published:** 2020-07-21

**Authors:** Dayle Sampson, Thomas D. Yager, Brian Fox, Laura Shallcross, Leo McHugh, Therese Seldon, Antony Rapisarda, Richard B. Brandon, Krupa Navalkar, Nandi Simpson, Sian Stafford, Eliza Gil, Cristina Venturini, Evi Tsaliki, Jennifer Roe, Benjamin Chain, Mahdad Noursadeghi

**Affiliations:** 1 Immunexpress, Seattle, WA USA; 2 Institute for Health Informatics, University College London, London, UK; 3 Division of Infection and Immunity, University College London, London, UK; 4 National Institute for Health Research University College London Hospitals Biomedical Research Centre, London, UK

**Keywords:** Blood transcriptional profiling, Bacterial infection, viral infection, Emergency department

## Abstract

**Background:**

There is an urgent need to develop biomarkers that stratify risk of bacterial infection in order to support antimicrobial stewardship in emergency hospital admissions.

**Methods:**

We used computational machine learning to derive a rule-out blood transcriptomic signature of bacterial infection (*SeptiCyte™ TRIAGE*) from eight published case-control studies. We then validated this signature by itself in independent case-control data from more than 1500 samples in total, and in combination with our previously published signature for viral infections (*SeptiCyte™ VIRUS*) using pooled data from a further 1088 samples. Finally, we tested the performance of these signatures in a prospective observational cohort of emergency department (ED) patients with fever, and we used the combined *SeptiCyte™* signature in a mixture modelling approach to estimate the prevalence of bacterial and viral infections in febrile ED patients without microbiological diagnoses.

**Results:**

The combination of *SeptiCyte™ TRIAGE* with our published signature for viral infections (*SeptiCyte™ VIRUS*) discriminated bacterial and viral infections in febrile ED patients, with a receiver operating characteristic area under the curve of 0.95 (95% confidence interval 0.90–1), compared to 0.79 (0.68–0.91) for WCC and 0.73 (0.61–0.86) for CRP. At pre-test probabilities 0.35 and 0.72, the combined *SeptiCyte™* score achieved a negative predictive value for bacterial infection of 0.97 (0.90–0.99) and 0.86 (0.64–0.96), compared to 0.90 (0.80–0.94) and 0.66 (0.48–0.79) for WCC and 0.88 (0.69–0.95) and 0.60 (0.31–0.72) for CRP. In a mixture modelling approach, the combined *SeptiCyte™* score estimated that 24% of febrile ED cases receiving antibacterials without a microbiological diagnosis were due to viral infections. Our analysis also suggested that a proportion of patients with bacterial infection recovered without antibacterials.

**Conclusions:**

Blood transcriptional biomarkers offer exciting opportunities to support precision antibacterial prescribing in ED and improve diagnostic classification of patients without microbiologically confirmed infections.

## Background

There is an urgent need to improve precision use of antibacterial drugs in order to minimise unnecessary prescribing [1]. This has a disproportionate impact within hospitals. In this setting, antibacterial overuse selects for drug-resistant bacteria and disrupts host-protective microbiota among individuals with increased risk of infection due to comorbidities, invasive procedures or instrumentation. All of this is compounded by exposure to drug-resistant pathogens from other hospital inpatients or the hospital environment [2–4].

Precision use of antibacterials is most challenging in emergency departments (ED), where assessments are based on a single time point with limited microbiological and laboratory data. Clinical features of severe sepsis unequivocally demand empirical antibacterials [5]. However, in patients who do not present with severe sepsis, better stratification of the risk of bacterial infection is expected to reduce antibacterial prescribing and may inform decisions about hospital admission, infection control practice and the choice of diagnostic investigations. These objectives have fuelled extensive efforts to identify biomarkers which discriminate bacterial and viral infections [6]. Importantly, routine diagnostic microbiology may provide inaccurate estimates of the true incidence of bacterial and viral infections in an ED setting. For example, in a prospective observational study, approximately 50% of suspected bacterial infections and 30% of suspected viral infections were not confirmed [7]. Accurate estimates of prior probability, needed to evaluate the predictive value of tests, are lacking. We hypothesise that molecular biomarkers of bacterial and viral infections may be used to obtain better estimates of the incidence of these infections in ED.

Blood leucocyte counts, C-reactive protein (CRP) and procalcitonin (PCT) are the most widely used biomarkers of infection used in current practice. Blood neutrophilia is associated with bacterial infection, but also occurs in response to trauma, seizures and vomiting [8]. Deficient neutrophil leucocytosis or leucopaenia is recognised in elderly patients with infection and in severe sepsis [9]. Lymphopaenia, sometimes associated with viral illnesses, is also reported as a correlate of bacteraemia [10]. Therefore, differential blood leucocycte counts have limited value as a biomarker to guide antibacterial use. In a multivariate analysis of clinical and laboratory parameters in febrile ED patients, elevated serum CRP and history of rigours were significantly associated with bacterial infection [11]. These were used in combination with serum PCT levels to develop a diagnostic risk score for bacterial infection, with a receiver operating characteristic (ROC) area under the curve (AUC) of 0.83 [11]. At a sensitivity of 95% and specificity of 32%, this risk score achieved a negative predictive value (NPV) of 73% compared to physician’s judgement which achieved 96% sensitivity, 50% specificity and 85% NPV. Even with suboptimal tests, the potential for biomarkers such as PCT to safely reduce initiation and continuation of antibacterial treatment has been demonstrated in selected ED patients [12]. In unselected adult ED patients with fever, a trial of PCT-guided treatment did not reduce antibacterial prescribing. This was partly attributed to physician non-adherence [13], vindicated by the fact that PCT only identified confirmed bacterial infections with ROC AUC of 0.68, underscoring the need for more accurate biomarkers.

In recent years, blood transcriptional profiling has emerged as a potentially powerful tool for diagnostic biomarker discovery in infectious diseases. We and others have focused this approach on identifying transcriptional signatures that discriminate between infective and non-infective inflammatory syndromes [14–16], and on discriminating between bacterial and viral infections [17–19]. Validation of these transcriptional signatures in prospective unselected ED cohorts is limited to two case-control studies: one of febrile children, in which a single gene-pair ratio achieved ROC AUC 0.97 for 28 confirmed bacterial infections compared to 23 confirmed viral infections [18], and our previously published validation of a transcriptional signature for viral infection (*SeptiCyte™ VIRUS*), in which the sum of two gene-pair ratios achieved ROC AUC 0.93 for 54 confirmed bacterial infections compared to 14 confirmed viral infections among febrile adults [19]. None has sought to compare the performance of transcriptional biomarkers to that of the existing biomarkers used almost ubiquitously in routine practice.

A key utility of a biomarker to support clinical decisions in ED is its potential use as a triage test to determine the risk of bacterial infection. In the present study, we describe the discovery and multi-cohort validation of a new blood transcriptomic signature (*SeptiCyte™ TRIAGE*) designed to be a “rule-out” test for bacterial infection. We then sought to benchmark the application of *SeptiCyte™ TRIAGE*, by itself and in combination with *SeptiCyte™ VIRUS*, against the performance of peripheral blood leukocytes and CRP to discriminate between confirmed bacterial and viral infections in unselected adults presenting to ED with fever. Finally, we used the combined signatures in a mixture modelling approach to estimate the incidence of bacterial and viral infections in patients from the same cohort with no microbiological diagnosis.

## Methods

### Selection of published data sets for discovery and validation of blood transcriptional signatures

We used four mutually exclusive groups of publicly available case-control data sets from GEO and ArrayExpress repositories that were of human origin and involved transcriptional profiling of whole blood or peripheral blood mononuclear cells without culture or stimulation. In the first group, we identified data sets derived from ED studies that included proven bacterial infections compared to uninfected healthy or virally infected controls (Additional Table S1). In the second group, we used data sets originally identified in our previous publication describing derivation and validation of the *SeptiCyte™ VIRUS* signature [19], in which neither cases nor controls included bacterial infection (Additional Table S2). In the third group, we identified all data sets that included proven bacterial infection cases and controls comprising healthy volunteers or patients with non-infective systemic inflammatory response syndrome (Additional Table S3). In the fourth group, we identified all remaining data sets, not included in any other group including proven bacterial infection cases and viral infection controls (Additional Table S5). The first two groups were identified by searches on 20 January 2015. The third and fourth groups were identified by searches on 17 May 2017.

### Study approval for prospective ED cohort

This study was approved by the UK National Research Ethics Service (reference: 10/H0713/51).

### ED study population and sampling

Consecutive adult patients presenting to University College London Hospitals Emergency Department service with a core temperature of > 37.5 °C were invited to participate (Table 1). Recruitment took place in 2010–2013, subject to availability of the recruitment team within regular working hours. All participants provided written informed consent. Where patients were unable to give consent directly, assent for their participation was sought from accompanying persons. In these cases, the patients’ consent to participate in the study was confirmed when patients were able to do so. Tempus™ tube (Fischer Scientific) blood samples were collected alongside routine blood tests in ED, within 4 h of presentation to hospital. Demographic, clinical laboratory results and clinical outcome data were obtained from the hospital electronic data repository. Blood RNA samples were not available for downstream analysis for a subset of the cohort either because the sample was not obtained at the time of recruitment or because the subsequent RNA extraction did not yield an adequate concentration of high-quality RNA (see Fig. 2 and Table 1).

**Table 1:** Selected characteristics of ED validation cohort

Characteristic		Proven bacterial infection		Proven viral infection		Empirical Abx (no positive micro)		Self-limiting illness (no positive micro)		Others	
		Total	Subset with RNA	Total	Subset with RNA	Total	Subset with RNA	Total	Subset with RNA	Total	Subset with RNA
Total number		102	68	16	14	176	93	32	25	6	0
Age (median, range)		54 (19–99)	51 (19–90)	45 (20–91)	44 (20–91)	52 (18–95)	46 (18–91)	46 (17–89)	37 (17–89)	41 (22–61)	n/a
Gender (% males)		52.6	51.5	42.9	35.7	52.8	48.4	51.9	48	66.7	n/a
Ethnicity (%)	White	72.2	64.7	50	50	66.9	62.4	55.6	40	16.7	n/a
	Asian	10.3	11.7	21.4	21.4	16.6	19.3	22.2	24	33.3	n/a
	African	7.2	5.8	28.6	28.6	6.1	4.3	14.8	12	33.3	n/a
	Others	10.3	17.6	0	0	10.4	13.9	7.4	24	16.7	n/a
Duration of illness (%)	< 2 days	25.0	27.9	18.8	14.2	26.3	30.1	22.6	24	16.7	n/a
	2–7 days	36.5	30.9	37.5	35.7	41.1	37.6	38.7	44	66.7	n/a
	7–14 days	7.7	5.9	18.8	0	9.1	9.7	9.7	4	0.0	n/a
	> 14 days	2.9	1.5	12.5	35.7	4.0	1.1	6.5	4	0.0	n/a
	Unknown	27.9	33.9	12.5	14.2	19.4	21.5	22.6	24	16.7	n/a
SIRS score (%)	0	2.0	1.5	6.3	7.1	4.5	5.4	18.8	8.0	33.3	n/a
	1	18.6	23.5	12.5	14.3	21.0	19.4	21.9	24.0	16.7	n/a
	2	38.2	33.8	68.8	64.3	38.1	39.8	43.8	52.0	16.7	n/a
	3	35.3	33.8	12.5	14.3	31.3	29.0	9.4	12.0	33.3	n/a
	4	5.9	7.4	0.0	0.0	5.1	6.5	6.3	4.0	0.0	n/a
Temperature (°C, range)		38.7 (37.5–40.6)	38.7 (37.5–40.6)	38.8 (37.7–40.1)	38.5 (37.7–40.1)	38.5 (37.5–40.9)	38.4 (37.5–40.9)	38.2 (37.5–39.4)	38.1 (37.5–39.4)	39.0 (37.9–40.4)	n/a
Systolic blood pressure mmHg (median, range)		130 (33–197)	125 (33–177)	122 (99–158)	115 (99–158)	130 (76–216)	130 (83–216)	127 (99–176)	128 (108–176)	123 (99–176)	n/a
Heart rate, beats/minute (median, range)		108 (63–170)	104 (70–149)	113 (80–150)	103 (80–147)	110 (55–173)	110 (68–173)	104 (54–148)	106 (69–148)	113 (90–137)	n/a
Respiratory rate, breaths/minute (median, range)		21 (14–54)	18 (14–40)	19 (14–24)	20 (14–24)	18 (13–48)	18 (14–48)	18 (14–40)	18 (15–14)	19 (16–20)	n/a
White cell count × 10^9^/mL (median, range)		12.8 (3.3–27.1)	13.3 (3.3–27.1)	8.0 (3.7–15.0)	7.8 (3.7–15.0)	12.0 (0.6–29.6)	12.0 (3.7–29.6)	10.1 (0.8–17.9)	10.5 (0.8–17.6)	8.1 (5.9–18.6)	n/a
Neutrophils (%)		83.4	80.8	76	75.6	83	81.9	77.7	78.1	79.1	n/a
CRP mg/L (median, range)		82.6 (3.9–476)	77.5 (3.9–375.6)	24.9 (3.9–146.7)	24.9 (3.9–146.7)	76.1 (0.7–512.6)	79.8 (0.7–290.2)	31.2 (0.6–282.5)	31.2 (0.6–282.5)	104.4 (9.9–233)	n/a
Antibacterial treatment (%)		100	100	56	64	100	100	0	0	33	n/a
Hospital length of stay (median no. of days, range)		6 (1–> 30)	6 (1–> 30)	3 (1–> 30)	3 (1–> 30)	4 (0–> 30)	4 (1–> 30)	2 (1–> 30)	2 (1–> 30)	3 (1–4)	n/a

### Clinical case definitions

Patients were classified into five separate groups based on laboratory microbiology and whether they received antimicrobial treatment during their hospital stay (Fig. 2, Table 1). Confirmed bacterial infection required culture of pathogenic bacteria from a sterile site (triggering initiation or continuation of antibacterial treatment). Confirmed viral infection required a positive viral PCR from a clinical specimen or serological evidence of acute infection. Those who had no positive microbiology were divided into two further groups on the basis of whether or not they received antimicrobial treatment. The final group consisted of microbiologically proven infection not due to bacterial and viral pathogens.

### Blood transcriptomic profiling

Samples from a subset of this cohort had previously been subjected to RNA sequencing (RNAseq) for validation of our previously published *SeptiCyte™ VIRUS* signature [19]. We complemented these data with targeted transcriptional profiling of all remaining samples from the study cohort for which adequate RNA was available, using customised NanoString nCounter assays (NanoString Technologies) [20]. Briefly, total blood RNA was extracted using the Tempus Spin RNA Isolation Kit (Ambion; Life Technologies). Sample signal values from this assay were background subtracted, normalised to the positive control (GAPDH expression value) in each run and log_2_-transformed. In order to ensure that we could pool RNAseq and Nanostring data, we undertook Nanostring profiling of a subset of samples already subjected to RNAseq, in order to make direct pairwise comparisons of the gene expression signatures used in the present study. This analysis showed high concordance with correlation coefficient of 0.99 (Fig. S1). Gene expression data used to calculate the blood transcriptional signature scores for the ED cohort are provided in Additional File 1.

### Data analysis

A blood transcriptional signature for bacterial infections (*SeptiCyte™ TRIAGE*) was derived from separate discovery and validation microarray data sets (Additional Table S1–3) using linear models of gene-pair ratios as described previously [14, 19] and in the “Results” section. The *SeptiCyte™* scores were calculated from log_2_-transformed gene expression values. For *SeptiCyte™ VIRUS*, the calculation comprised (ISG15 + OASL) − (IL16 + ADGRE5). For *SeptiCyte™ TRIAGE*, the calculation comprised (DIAPH2 + GBP2 + TLR5) − (IL7R + GIMAP4 + FGL2). The combined *SeptiCyte™* score was calculated by the subtracting the *SeptiCyte™ VIRUS* score from the *SeptiCyte™ TRIAGE.*

Unit variance scaling of gene expression in multi-cohort data (Table S5) was performed by subtracting the mean and dividing by the standard deviation in each data set [21]. Mann-Whitney and receiver operating characteristic (ROC) analyses were performed in GraphPad Prism v6. The Youden index of ROC curves was calculated from the sum of sensitivity and specificity − 1. Bayesian conditional probabilities were calculated as previously described [22]. Ninety-five per cent confidence intervals are provided for each measure of test performance. We used mixture modelling to estimate the proportions of bacterial and viral infections in patients recruited to the ED fever cohort. The frequency distributions for *SeptiCyte™* scores for cases of proven bacterial and viral infections were fitted to two normal distributions using maximum likelihood. The posterior probabilities for these two classes were used to estimate the relative likelihood of a bacterial or viral diagnosis for a given value of *SeptiCyte™* score. New distributions were then constructed by mixing the two distinct normal distributions in different proportions of viral to bacterial cases (ranging 0.1 to 10 in steps of 5 × 10^−4^) using the *R* function rnorm() to generate the appropriate set of random deviates. Each mixed distribution was compared to the empirical distributions for cases of unknown aetiology. The difference between predicted and observed distribution was measured with the Jensen-Shannon divergence using CalcJSDivergence() in the *R* package textmineR. The proportion giving the minimum divergence was chosen as the best fit.

In silico discovery of the *SeptiCyte™* signatures was performed by Immunexpress. No a priori sample size calculation was performed for recruitment of the ED cohort. The evaluation of the performance of this signature in the prospective ED cohort was conducted entirely by independent investigators at UCL, with no commercial interest in Immunexpress. This includes the design of the inclusion criteria for the cohort study, participant recruitment, clinical data collection and case ascertainment, sample collection, measurement of the RNA signatures and evaluation of the performance of these signatures. The Standards for Reporting Diagnostic accuracy studies (STARD) checklist is available as an online supplement.

## Results

### Discovery and in silico validation of a blood transcriptional signature associated with bacterial infections (*SeptiCyte™ TRIAGE*)

**Fig. 1:**
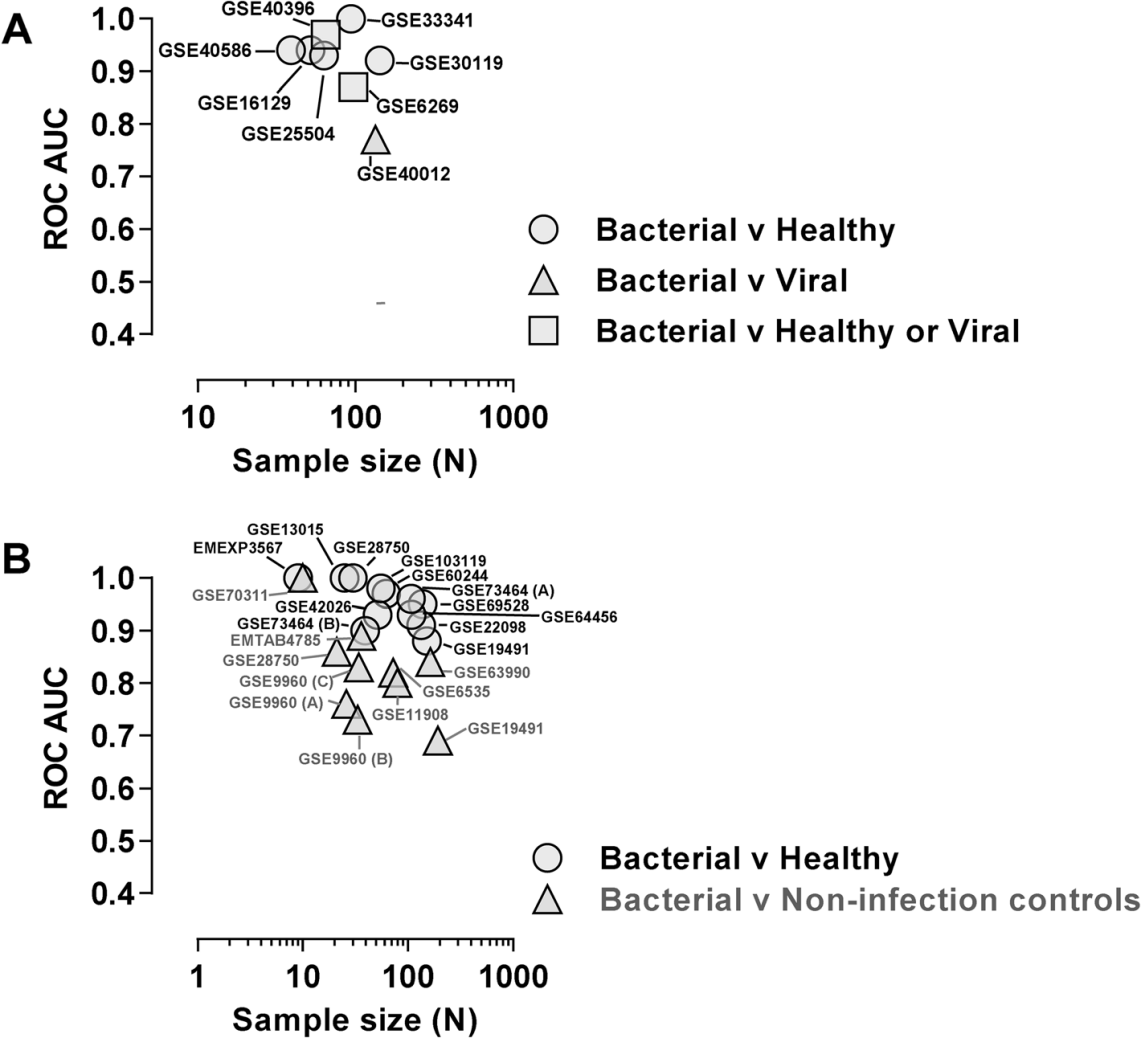
In silico discovery and validation of *SeptiCyte™ TRIAGE*. Receiver operating characteristic area under the curve (ROC AUC) for discriminating between bacterial infection and the different control groups (indicated) using the SeptiCyte™ TRIAGE score, and study sample size for publicly available data sets used for (**a**) discovery and (**b**) validation. Each study is identified by the corresponding Gene Expression Omnibus (GEO) accession number label for the data points. Additional information about the discovery data sets is provided in Table S2, and for the validation data sets, it is provided in Table 2

We sought to identify a parsimonious blood transcriptional signature for bacterial infection using similar computational approaches to derive gene signatures for sepsis in the ICU setting [14] and for viral infections in general [19]. Eight public microarray data sets comparing patients with bacterial infections to controls (Additional Table S1) were used to discover gene-pair ratios that were differentially expressed (with false discovery rate < 0.01) and that discriminated between bacterial and control cases with ROC AUC > 0.7 in each data set. We then sought to exclude non-specific biomarkers of disease, by identifying and excluding all gene-pair ratios that discriminated non-infective diseases from their controls with ROC AUC > 0.8 (in blood transcriptomic data from eight published data sets of non-infective diseases; Additional Table S2). Finally, in the pool of eight normalised discovery data sets, we used stepwise addition of the remaining gene-pair ratios ranked by greedy forward selection to maximise the mean ROC AUC between bacterial cases and controls. This approach identified a blood transcriptional signature, *SeptiCyte™ TRIAGE*, based on the sum of three gene-pair ratios (DIAPH2/IL7R, GBP2/GIMAP4, TLR5/FGL2), which differentiated bacterial infections from viral infections and healthy controls in the discovery data sets (Table S1) with a ROC AUC range of 0.77–1 (Fig. 1a). We then sought to validate this signature in independent published data sets derived from patients with bacterial infection and healthy volunteers, or non-infective diseases (Fig. 1b and Additional Table S3). The *SeptiCyte™ TRIAGE* signature discriminated bacterial infection cases from healthy volunteers with a ROC 0.70–1.

### Using SeptiCyte TRIAGE to discriminate bacterial and viral infections in adult ED patients with a fever

**Fig. 2:**
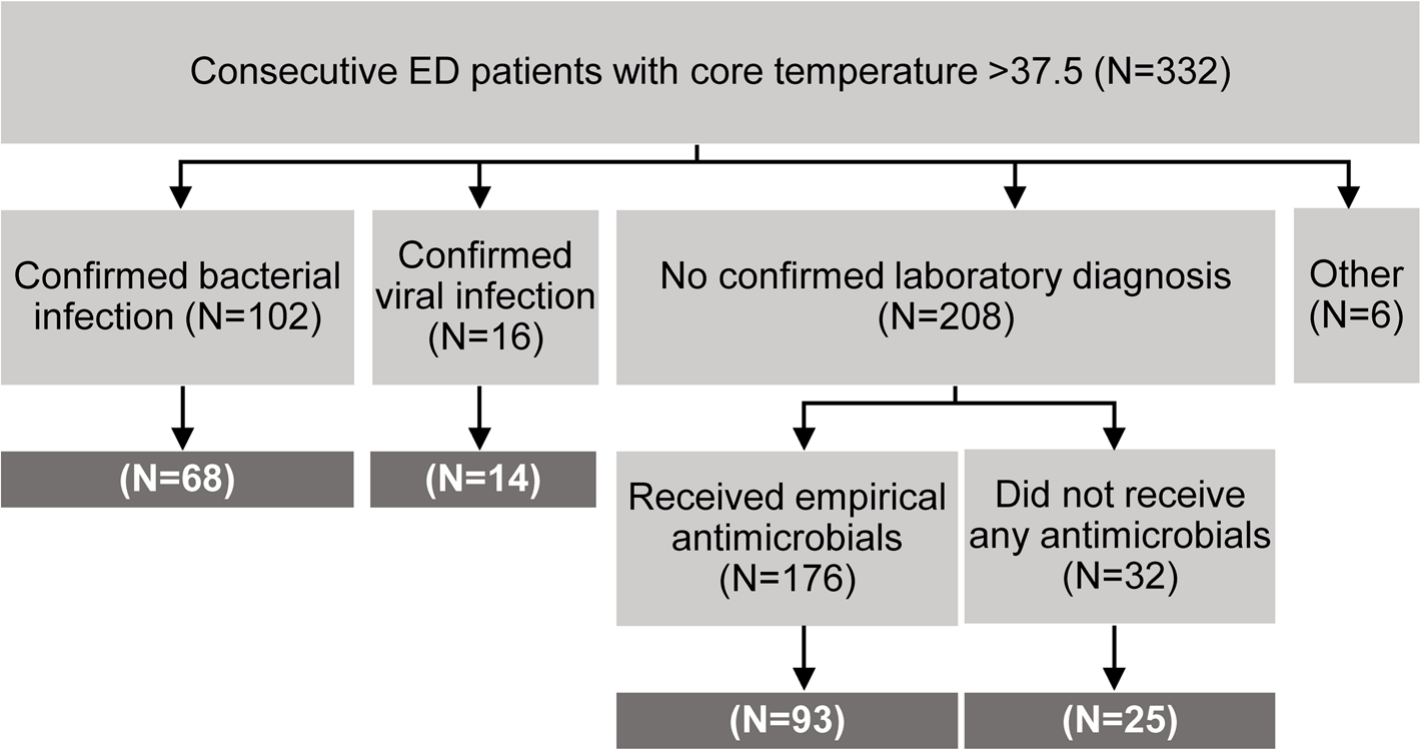
Consort diagram for FEVER study. In this study, 332 patients with fever were enrolled, of which 104 had confirmed bacterial infection, 16 had confirmed viral infection, 206 had no microbiologically confirmed laboratory diagnosis and six had non-bacterial and non-viral infections. Of those patients with no microbiologically confirmed laboratory diagnosis, 175 received antimicrobials and 31 did not. Numbers in white indicate the samples for which blood transcriptional profiles were available

Next, we sought to validate the *SeptiCyte™ TRIAGE* signatures in the ED setting, and to benchmark the performance of these signatures against peripheral blood leukocyte counts and CRP, used almost universally in ED. We recruited an observational cohort of 332 consecutive patients presenting to the ED in a large UK teaching hospital with a temperature of > 37.5°, for whom we were able to obtain consent from the patient or where necessary the next of kin (Table 1, Fig. 2, Additional Fig. S2). The patients ranged from 17 to 99 years of age and 48% were male. No predefined risk factors (Additional Table S4) for infection were evident in 147 (44%) patients in the cohort. Of the remainder, most had one risk factor (Additional Fig. S2C-D). Recruitment to the study did not affect the diagnostic investigations or management of the participants in any way. Hence, the diagnostic yield and use of antimicrobial treatment in this cohort reflected routine practice in a UK setting. Confirmatory microbiological diagnosis became available for 124 patients (38%), including 102 bacterial and 16 viral infections, four cases of malaria, one attributed to fungal infection and one to *Entamoeba histolytica* (Additional Fig. S2E). Of the 208 cases with no positive microbiology, 32 recovered without receiving antimicrobials. The remaining 176 received empirical antibacterial treatment (Fig. 2). Blood transcriptomic data were available on 68 patients with proven bacterial infection, 14 patients with proven viral infection and 118 patients with no confirmed laboratory diagnosis of infection of whom 93 received empirical antibacterial treatment (Fig. 2).

**Fig. 3:**
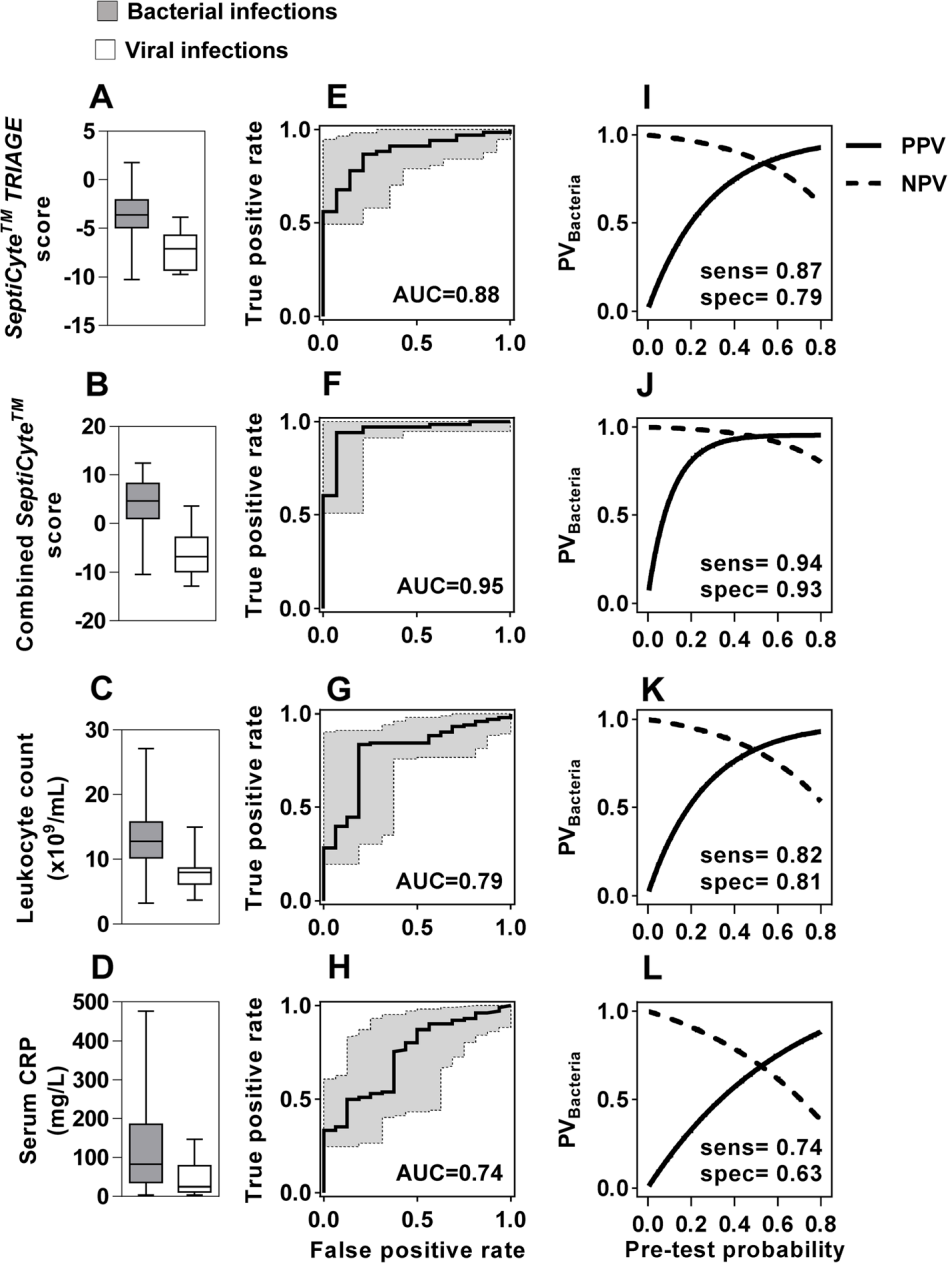
*SeptiCyte™ TRIAGE* score, combined *SeptiCyte™* score, blood leukocyte count and serum C-reactive protein in the ED fever cohort. Distributions of *SeptiCyte™ TRIAGE* score (**a**), combined *SeptiCyte™* score (**b**), peripheral blood leukocyte count (**c**) and serum C-reactive protein (CRP) (**d**) for proven bacterial and viral infections. Receiver operating characteristic area under the curve (ROC AUC ± 95% confidence intervals) for discrimination of proven bacterial and viral infections using *SeptiCyte™ TRIAGE* score (**e**), combined *SeptiCyte™* score (**f**), peripheral blood leukocyte count (**g**) and serum C-reactive protein (CRP) (**h**). Positive predictive value (PPV) and negative predictive value (NPV) of bacterial infection at different pre-test probabilities using the sensitivity and specificity derived from the maximal Youden index of the ROC curves for each of the *SeptiCyte™ TRIAGE* score (**i**), combined *SeptiCyte™* score (**j**), peripheral blood leukocyte count (**k**) and serum C-reactive protein (CRP) (**l**)

Within this ED fever cohort, the *SeptiCyte™ TRIAGE* score for 68 patients with confirmed bacterial infection and 14 with confirmed viral infection was derived from RNAseq data. The *SeptiCyte™ TRIAGE* score was significantly higher in bacterial infection compared to viral infection (Fig. 3a) and achieved a ROC AUC of 0.88 (0.81–0.97) (Fig. 3e). We used the ROC curve Youden index to identify the threshold value providing the maximum classification accuracy for a given test. At this threshold, the *SeptiCyte™ TRIAGE* score achieved a sensitivity of 0.87 (0.76–0.94) and specificity of 0.79 (0.5–0.95), which we then used to calculate the positive and negative predictive values for this test, across a range of pre-test probabilities (Fig. 3i). Assuming prior probabilities of 72% or 35% for upper bound and lower bound of bacterial infection in febrile ED patients [7], the NPV of the *SeptiCyte™ TRIAGE* score at its Youden index was calculated to be 0.70 (0.45–0.80) and 0.92 (0.79–095) respectively (Fig. S3).

### Combining SeptiCyte™ TRIAGE and SeptiCyte™ VIRUS to obtain greater discrimination

Next, we tested the hypothesis that combining our tests for viral (*SeptiCyte™ VIRUS*) [19] and bacterial (*SeptiCyte™ TRIAGE*) infection would achieve better discrimination between cases of bacterial and viral infection. This hypothesis was based on the premise that because each signature was independently derived to discriminate between different classes (bacterial infection from controls in the case of *SeptiCyte™ TRIAGE*, and viral infections from controls in the case of *SeptiCyte™ VIRUS*), each signature would reflect different or orthogonal features of the cases and hence, in combination, would offer better discrimination between bacterial and viral infections than either signature alone. In order to test this assumption, we first pooled publicly available data (following unit variance scaling), from 1088 bacterial and viral infections in twelve case-control studies that had not contributed to any of the discovery data for either signature (Additional Table S5). Comparison of the two scores in these data revealed a statistically significant inverse correlation, but an *R*^2^ coefficient of only 0.13, indicating that the majority of the signal from each score was orthogonal (Fig. S3A). Consistent with our hypothesis, a combined score, generated by subtracting the viral score from the bacterial score, was found to discriminate bacterial and viral infections in these pooled data with a ROC AUC of 0.88 (0.86–0.9), compared to the *SeptiCyte™ TRIAGE* alone (ROC AUC 0.76, 0.73–0.79) or *SeptiCyte™ VIRUS* score alone (ROC AUC of 0.84, 0.82–0.87) (Fig. S3B).

This analysis provided independent validation of the combined *SeptiCyte™* score in case-control data, but our primary aim was to test its performance in the observational ED fever cohort. In this setting, the distribution of values for the combined score was significantly higher in bacterial infections compared to viral infections (Fig. 3b) and discriminated between the two groups with a ROC AUC of 0.95 (0.90–1) (Fig. 3f). At the Youden index of the ROC curve, the combined score achieved a sensitivity of 0.94 (0.86–0.98) and specificity of 0.93 (0.66–0.99) for bacterial infections. At this threshold, the PPV and NPV of the combined score are shown across the range of pre-test probabilities in Fig. 3j. Assuming prior probabilities of 72% or 35% for upper bound and lower bound of bacterial infection in febrile ED patients [as estimated in reference # 7], the NPV of the combined *SeptiCyte™* score at its Youden index was calculated to be 0.86 (0.64–0.96) and 0.97 (0.90–0.99) respectively (Additional Fig. S4).

Peripheral blood leucocytosis and high CRP are frequently used as biomarkers of bacterial infection in routine clinical practice in the ED. Although there were statistically significant correlations between the combined *SeptiCyte™* scores and leukocyte count or CRP, the correlation coefficients were weak (*R*^2^ of 0.3 leukocyte count and 0.07 for CRP, Additional Fig. S5). The distribution of leucocyte counts and CRP measurements were statistically higher in patients with bacterial infections compared to those with viral infections. Discrimination of these cases yielded ROC AUC of 0.79 (0.68–0.91) for WCC and 0.73 (0.61–0.86) for CRP. At the Youden index of these ROC curves, the PPV and NPV of each test are shown across the range of pre-test probabilities in Fig. 3k and l. At their Youden indices, the NPV of these measurements at an estimated prior probability of bacterial infection 35% [7] was calculated to be 0.90 (0.80–0.94) for WCC and 0.88 (0.69–0.95) for CRP. At an estimated prior probability of 72% [7], the NPV reduced to 0.66 (0.46–0.79) for WCC and 0.60 (0.32–0.72) for CRP (Fig. S4). On the basis that a test used to rule-out bacterial infection must achieve high NPV even at relatively high prior probability of bacterial infection, our data show that the combined *SeptiCyte™* score outperforms WCC and CRP.

In a sensitivity analysis, we also calculated the NPV for each of these tests using the Youden index thresholds to exclude bacteraemia within our ED cohort. The combined *SeptiCyte™* score achieved a NPV of 1.0 (0.94–1.0) compared to 0.94 (0.86–0.97) for CRP and 0.89 (0.80–0.94) for WCC. The performance metrics of all these tests are presented side-by-side in Table 2.

**Table 2:** Summary performance metrics for *SeptiCyte™* scores, WCC and CRP for discrimination of proven bacterial and viral infections in the ED cohort, at the Youden index threshold for each test

	***SeptiCyte™ TRIAGE***	Combined ***SeptiCyte™***	WCC	CRP
**ROC AUC**	0.89 (0.81–0.97)	0.95 (0.90–1)	0.79 (0.68–0.91)	0.73 (0.61–0.86)
**Sensitivity**	0.87 (0.76–0.94)	0.94 (0.86–0.98)	0.84 (0.75–0.90)	0.87 (0.79–0.93)
**Specificity**	0.79 (0.5–0.95)	0.93 (0.66–0.99)	0.81 (0.54–0.96)	0.5 (0.25–0.75)
**LR**^**+ve**^	4.04 (1.50–19.48)	13.18 (2.53–546.5)	4.45 (1.64–22.24)	1.75 (1.05–3.77)
**LR**^**−ve**^	0.17 (0.10–0.48)	0.06 (0.02–0.22)	0.20 (0.10–0.46)	0.25 (0.09–0.84)
*At prior probability of bacterial infection of 35%*				
**PPV**_**bacteria**_	0.66 (0.34–0.91)	0.88 (0.51–0.99)	0.70 (0.39–0.93)	0.35 (0.15–0.62)
**NPV**_**bacteria**_	0.70 (0.45–0.80)	0.86 (0.64–0.96)	0.90 (0.80–0.94)	0.88 (0.69–0.95)
*At prior probability of bacterial infection of 72%*				
**PPV**_**bacteria**_	0.91 (0.79–0.98)	0.97 (0.87–0.99)	0.92 (0.80–0.98)	0.82 (0.73–0.91)
**NPV**_**bacteria**_	0.92 (0.79–095)	0.97 (0.90–0.99)	0.66 (0.46–0.79)	0.60 (0.32–0.72)

Values in brackets represent the 95% confidence intervals. *LR*^*+ve*^ likelihood ratio of bacterial infection for a positive result, *LR*^*−ve*^ likelihood ratio of bacterial infection for a negative result, *PPV*_*bacteria*_ positive predictive value for bacterial infection, *NPV*_*bacteria*_ negative predictive value for bacterial infection

### Estimating the true prevalence of bacterial and viral infections in the ED.

The study by Limper et al. [7] highlights that even when microbiological investigations are optimised, estimates of the prevalence of bacterial infection ranged from 35 to 72% [7]. In this setting, diagnostic biomarkers may offer more accurate estimates of the prevalence of bacterial and viral infections and consequently more accurate estimates of the predictive value of a test.

**Fig. 4:**
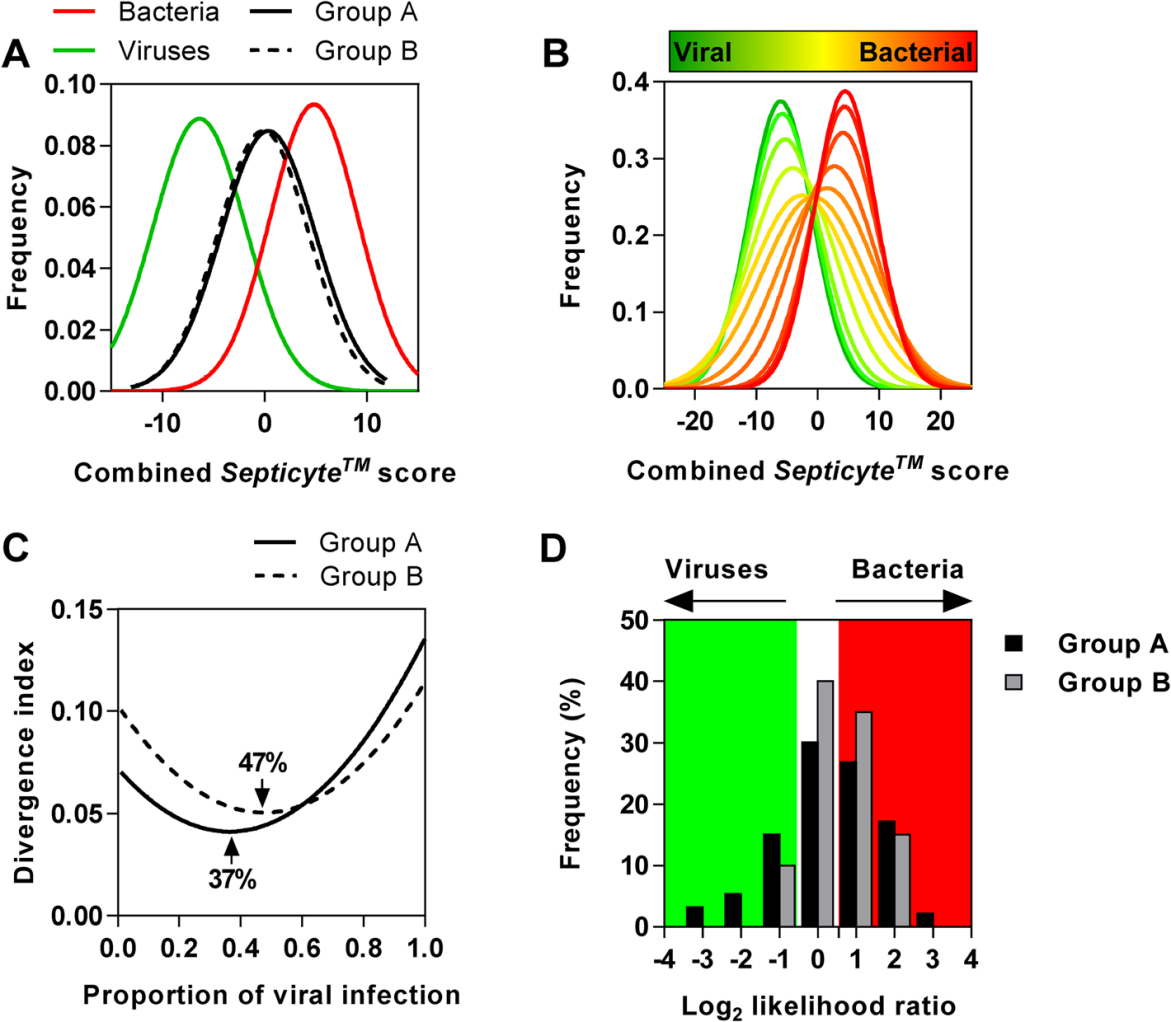
Estimation of bacterial and viral infection rates in different ED patient groups with a fever. **a** Frequency distributions of the combined (*SeptiCyte™ TRIAGE* and *SeptiCyte™ VIRUS*) score in ED fever cohort cases with proven bacterial and viral infections and in cases with no microbiological diagnosis who received empirical antibacterial treatment (group A) or recovered without antibacterial treatment (group B). **b** Computationally generated distributions generated by mixing different proportions of the two distinct distributions of combined *SeptiCyte™* scores from cases of proven bacterial and viral infections shown in (**a**). **c** The Jensen-Shannon divergence index, used to identify the distribution in (**b**) that was most similar to the distributions of group A and group B shown in) (**a**), giving an estimate of the proportion of viral infections in each group indicated. **d** The relative likelihood of each individual case in group A and group B being derived from the distribution of combined *SeptiCyte™* scores associated with proven bacterial or viral infections

We used the combined *SeptiCyte™* score to infer the classification of cases within our ED fever cohort which did not yield positive microbiological results, using Gaussian mixture modelling [23]. We divided the 119 available whole blood RNA samples from these cases into 93 cases that received empirical antibacterials (group A) and 26 cases that experienced self-limiting illnesses without any antibacterials (group B). The frequency distribution of the combined *SeptiCyte™* score for both these groups was compared to that of proven bacterial and viral infections from the same cohort (Fig. 4a). We fitted a normal distribution to the known bacterial and viral distributions, and then calculated predicted frequency distributions which would be observed for cohorts containing different proportions of bacterial and viral cases (Fig. 4b). We compared the observed distribution of scores in groups A and B to the predicted distributions and estimated the proportion of viral infection cases which showed the best fit to the data by minimising the Jansen-Shannon divergence [24] between predicted and observed distributions (Fig. 4c). This analysis estimated that 37% of patients who received empirical antibacterials were classified by the combined *SeptiCyte™* score as viral infections, compared to 47% of patients who did not receive antibacterials. Assuming all 208 febrile ED patients without microbiological diagnosis had either bacterial or viral infection, our analysis suggests that 229 (69%) of the total cohort of 332 had bacterial infections of which 45% were microbiologically proven, and 97 (30%) had viral infections of which 16.5% had laboratory confirmation. Under the reasonable assumption that not all febrile illnesses will be exclusively due to bacterial and viral infections, these estimates represent the upper limits of the prevalence of bacterial and viral infections in ED.

Finally, we estimated the relative likelihood of having a bacterial versus viral infection in individual cases. We used the fitted distributions for the cohorts of proven bacterial and viral infections to estimate the posterior probability for each data point in patients without positive microbiology that either received empirical antibacterial treatment (group A) or recovered without antibacterials (group B). This value provided an estimate of the relative likelihood of having a bacterial or viral infection for a given combined *SeptiCyte™* score. Approximately 70% of group A patients could be classified as bacterial or viral infection with greater than a two-fold likelihood ratio, and about 60% of group B patients could be classified as bacterial or viral infection with greater than a two-fold likelihood ratio (Fig. 4d). In this analysis, 24% of patients who received empirical antibacterials had greater than two-fold likelihood of having had a viral infection and 40% of patients who recovered without receiving antibacterials had more than two-fold likelihood of having had a bacterial infection.

## Discussion

We describe a novel blood transcriptomic signature specific for bacterial infection (*SeptiCyte™ TRIAGE*), which we validate using data from 1575 samples in a multi-cohort analysis of published case-control studies. We combined this signature with our previously published transcriptomic signature for viral infections (*SeptiCyte™ VIRUS*) and validated the application of the combined signature in published case-control data from 1088 samples and in a further independently derived cohort of emergency adult admissions to hospital. In this cohort, the combined signature score achieved a ROC AUC of 0.95 for discriminating between proven bacterial and viral infections. Peripheral blood leukocyte and CRP measurements, which remain the cornerstone of early diagnostic biomarkers to guide the use of antibacterial drugs, only achieved ROC AUCs of 0.79 and 0.74 respectively. In the present study, we were not able to make comparison with PCT because this is not used routinely in adult ED settings in the UK.

In our ED cohort, the combined *SeptiCyte™* score achieved an NPV of 0.86–0.97 across the range of prior probabilities for bacterial infection within febrile ED patients. On the basis that prolonged delay in antimicrobial treatment for severe sepsis is associated with increased mortality [25, 26], we propose that the imperfect sensitivity of any such biomarker means that its application as a rule-out test, to reduce empirical antimicrobial prescriptions in ED, will be restricted to patients with non-severe illness. Even so, the effectiveness of this application may be sensitive to heterogeneity in clinician assessments of risk/benefit ratio for individual patients. Of note, within the present data set, the combined *SeptiCyte™* score achieved 100% NPV for bacteraemia, suggesting that such an approach may in fact provide an effective rule-out test for severe bacterial infection. In addition, as a quantitative test, the combined *SeptiCyte™* score does not necessarily require a specific threshold to provide a binary result. Clinicians may wish to consider the sensitivity, specificity and predictive value of different test thresholds, depending on their tolerance for false positive or false negative results.

The major limitation of our study is the relatively small sample size of proven bacterial and viral infections in our ED cohort. Notwithstanding the need for extended validation in larger sample sizes, our data encourage the further development of blood transcriptomic signatures for rapid near-patient tests to support the differential diagnosis of bacterial and viral infections. In addition to the technological development required to realise their potential, further evaluation of factors that may confound the performance of gene expression signatures is necessary. Specifically, the range of non-infectious diseases, or non-viral and non-bacterial infections that may modulate these transcripts, and the impact of time on antibacterial or antiviral treatment should be examined further. It is particularly important to establish the window of opportunity in which these measurements can be used to reliably distinguish between infections, or to evaluate their potential role in monitoring the response to treatment. Also of note, our approach to discovery of the most concise biosignature is agnostic to the biological function of the genes and precludes confident inferences about the functional pathways represented by these signatures. Such inferences are statistically dependent on identification of multiple components of a pathway, and our statistical power to identify the associated pathway is substantially reduced by selecting the minimum number of genes required to achieve the maximum classification accuracy.

We hypothesised that blood transcriptional biomarkers that accurately discriminate between bacterial and viral infections may offer an opportunity to obtain better estimates of the true incidence of these two classes of infectious disease. Such epidemiological data are critical to our ability to incorporate prior probabilities in clinical assessments, and our interpretation of diagnostic laboratory tests. In the cohort of adult ED fever patients recruited in this study, we estimated that an upper limit of the prevalence of bacterial infection to be 69%, in keeping with the total proportion of cases attributed to bacterial infection in a similar ED cohort [7], but a higher proportion of viral infections suggesting that viral illnesses are substantially underdiagnosed. In our study, 206 (62%) had no microbiological diagnosis. Of those that received empirical antibacterial treatment, the application of mixture modelling using the combined *SeptiCyte™* score estimated that 24% had more than two-fold likelihood of being due to a viral infection, suggesting that these patients may not have needed antibacterial drugs. These patients may also be targeted for enhanced virological testing and may merit source isolation to mitigate against onward transmission of viral infection.

Interestingly, in ED fever patients who had no positive microbiology, but fully recovered without antibacterial treatment, mixture modelling using the combined *SeptiCyte™* score classified that as much as 40% had more than two-fold likelihood of being due to bacterial infection. These data support the concept that some bacterial infections may be self-limiting and do not necessarily need antibacterial treatment. Hence, any policy for antibacterial prescribing triggered exclusively by diagnostic biomarkers for bacterial infection may inadvertently increase unnecessary antibacterial use.

Importantly, 30% of microbiologically undiagnosed cases that received empirical antibacterials and 40% of those that did not receive antibacterial treatment could not be classified using the combined *SeptiCyte™* score with greater than two-fold likelihood ratio. A plausible explanation in some cases may be the presence of co-infection or a non-infective cause of fever, but there may be many additional potential confounders. Further investigation is required using larger studies with sufficient power to evaluate the possible effects of age, gender, ethnicity, comorbidities and immunomodulatory drugs. Ultimately, integration of clinical and laboratory data will be required to derive models which quantitate the risk of bacterial infections which do or do not require antibacterial treatment.

## Conclusions

Our study supports the development of blood transcriptomic signatures for rapid near-patient tests to discriminate between bacterial and viral infections in ED. We expect this approach may inform precision use of antibacterials, but also infection control practice and better use of targeted diagnostic tests for bacterial and viral infection.

## Supplementary information

**Additional file 1: Figure S1.** Comparison of Nanostring and RNAseq derived blood transcriptional signature scores. **Figure S2.** Demographic and microbiological summary of the ED fever cohort. **Figure S3.** Comparison of SpeticyteTM TRIAGE, SepticyteTM VIRUS and combined SpeticyteTM scores in pooled case-control data of bacterial and viral infections. **Figure S4.** Negative predictive value of different biomarkers for identification of ED patients with proven bacterial infection. **Figure S5.** Comparison of peripheral blood leukocyte count and C reactive protein levels with blood transcriptional biomarkers.

**Additional file 2: Table S1.** GEO Datasets Used for Discovery of SeptiCyte™ Triage. **Table S2.** GEO Datasets used to test specificity of differentially expressed gene pair ratios for discovery of the SeptiCyteTM TRIAGE signature. **Table S3.** GEO Datasets used to validate the SeptiCyte™ TRIAGE signature. Table S4. Apriori defined list of risk factors for infection. Table S5. GEO Datasets used to validate the combined SeptiCyteTM signature.

**Additional file 3: **Spreadsheet for anonymised clinical metadata *Septicyte™* scores, WCC and CRP measurements for all study participants.

## Data Availability

All data generated by this study are included in this published article and its supplementary information files, or available in public repositories under the accession numbers provided.

## Abbreviations

CI: Confidence interval
CRP: C-reactive protein
ED: Emergency department
GEO: Gene Expression Omnibus
NPV: Negative predictive value
PCT: Procalcitonin
PPV: Positive predictive value
RNAseq: RNA sequencing
ROC AUC: Receiver operating characteristic area under the curve
