# Correction to: Blood transcriptomic discrimination of bacterial and viral infections in the emergency department: a multi-cohort observational validation study

**DOI:** 10.1186/s12916-020-01756-x

**Published:** 2020-09-12

**Authors:** Dayle Sampson, Thomas D. Yager, Brian Fox, Laura Shallcross, Leo McHugh, Therese Seldon, Antony Rapisarda, Roslyn A. Hendriks, Richard B. Brandon, Krupa Navalkar, Nandi Simpson, Sian Stafford, Eliza Gil, Cristina Venturini, Evi Tsaliki, Jennifer Roe, Benjamin Chain, Mahdad Noursadeghi

**Affiliations:** 1 Immunexpress, Seattle, WA USA; 2 Institute for Health Informatics, University College London, London, UK; 3 Division of Infection and Immunity, University College London, London, UK; 4 National Institute for Health Research University College London Hospitals Biomedical Research Centre, London, UK

**Correction to: BMC Med 18, 185 (2020)**

**https://doi.org/10.1186/s12916-020-01653-3**

Following publication of the original article [1], it was brought to our attention that the member of the collaborating team at Immunexpress Roslyn A. Hendriks, had initially been omitted from the authorship of the article due to an oversight.

Roslyn A. Hendriks was involved in the original conception of the Septicyte TRIAGE signature, but had since left the Immunexpress team. The authors feel that her intellectual contribution to the development of this signature merits her inclusion in the authorship of the article.

The full and correct authorship has been updated in the original article and included in the author list of this Correction.

The ‘Authors’ contributions’ section is also corrected to read: DS, TY, RB, RH and MN conceived and designed the study. NS, SS, EG, ET and JR performed sample processing and/or data collection. DS, BF, LS, LM, TS, AR, KN, CV, BC and MN performed the data analysis. DS, TY and MN wrote the manuscript with input from all the authors. All authors read and approved the final manuscript.
