# Supplementary material for: Blood transcriptomic discrimination of bacterial and viral infections in the emergency department: a multi-cohort observational validation study
Source: BMC Med. 2020 Jul 21;18:185. doi: 10.1186/s12916-020-01653-3 (PMC7372897; doi:10.1186/s12916-020-01653-3)
Supplement: Supplementary file 1 — Additional file 1: Figure S1. Comparison of Nanostring and RNAseq derived blood transcriptional signature scores. Figure S2. Demographic and microbiological summary of the ED fever cohort. Figure S3. Comparison of SpeticyteTM TRIAGE, SepticyteTM VIRUS and combined SpeticyteTM scores in pooled case-control data of bacterial and viral infections. Figure S4. Negative predictive value of different biomarkers for identification of ED patients with proven bacterial infection. Figure S5. Comparison of peripheral blood leukocyte count and C reactive protein levels with blood transcriptional biomarkers. [file 12916_2020_1653_MOESM1_ESM.docx]

### **Figure S1**

**Comparison of Nanostring and RNAseq derived blood transcriptional signature scores.**

### **Figure S2**

A

B

**C**

**D**

**E**

***Demographic and microbiological summary of the ED fever cohort.***(A) Age distribution of patients in the ED fever cohort. (B) Infections represented within the ED fever cohort classified by the physiologiocal system affected (numbers represent percentages). (C) Numbers of patients with predefined risk factors for infection, and (D) the relative proportions (%) of the most frequent risk factors. (E) Relative proportions microbiological diagnoses.

Figure S3

**A**

**B**

***Comparison of Speticyte^TM^ TRIAGE, Septicyte^TM^ VIRUS and combined Speticyte^TM^ scores in pooled case-control data of bacterial and viral infections.* (A)** Correlation of *Speticyte^TM^ TRIAGE* and *Septicyte^TM^ VIRUS* scores in 1088 pooled data from 12 studies (504 case of bacterial infection and 584 cases of viral infection). **(B)** ROC analysis of each signature for correct classification of bacterial viral infection cases (ROC AUC and 95% confidence intervals shown in brackets).

****Figure S4

**72% prior probability of bacterial infection**

**35% prior probability of bacterial infection**

**A**

**B**

***Negative predictive value of different biomarkers for identification of ED patients with proven bacterial infection.*** Negative predictive value (NPV) with 95% confidence intervals of each of the blood biomarkers indicated across a range of threshold values assuming (A) 35% or (B) 72% prior probability using Bayesian conditional probabilities. Dashed lines indicate the Youden Index threshold from the ROC curve (Figure 3 of the main manuscript) of each biomarker.

Figure S5

**A**

**B**

***Comparison of peripheral blood leukocyte count and C‑reactive protein levels with blood transcriptional biomarkers.* (A)** Pairwise comparison of peripheral blood leukocyte count and **(B)** C‑reactive protein (CRP) levels with the combined *Septicyte^TM^* scores, showing r^2^ and the p values for the linear regression between each pair of variables.
