# Supplementary material for: Blood transcriptomic discrimination of bacterial and viral infections in the emergency department: a multi-cohort observational validation study
Source: BMC Med. 2020 Jul 21;18:185. doi: 10.1186/s12916-020-01653-3 (PMC7372897; doi:10.1186/s12916-020-01653-3)
Supplement: Supplementary file 2 — Additional file 2: Table S1. GEO Datasets Used for Discovery of SeptiCyte™ Triage. Table S2. GEO Datasets used to test specificity of differentially expressed gene pair ratios for discovery of the SeptiCyteTM TRIAGE signature. Table S3. GEO Datasets used to validate the SeptiCyte™ TRIAGE signature. Table S4. Apriori defined list of risk factors for infection. Table S5. GEO Datasets used to validate the combined SeptiCyteTM signature. [file 12916_2020_1653_MOESM2_ESM.docx]

#### Table S1. GEO Datasets Used for Discovery of SeptiCyte^TM^ Triage

| **Dataset accession no** | **Study population** | **Cases** | **N** | **Controls** | **N** |
| --- | --- | --- | --- | --- | --- |
| GSE30119 | Paediatric | *Staphylococcus aureus* | 99 | Healthy | 44 |
| GSE33341 | Adult | *Staphylococcus aureus*, *Escherichia coli* | 51 | Healthy | 43 |
| GSE16129 | Paediatric | *Staphylococcus aureus* | 42 | Healthy | 10 |
| GSE25504 | Neonatal | Bacterial | 26 | Healthy | 37 |
| GSE40586 | Adult | Bacterial meningitis | 21 | Healthy | 18 |
| GSE6269 | Paediatric | *Staphylococcus aureus*  *Streptococcus pneumoniae*  *Escherichia coli* | 73 | Healthy / Influenza | 24 |
| GSE40012 | Adult | Bacterial pneumonia | 19 | Viral infection | 115 |
| GSE40396 | Paediatric | Bacterial | 8 | Viral or Healthy | 57 |
|  | | Total | 339 |  | 348 |

#### Table S2. GEO Datasets used to test specificity of differentially expressed gene pair ratios for discovery of the SeptiCyte^TM^ TRIAGE signature

| **Dataset accession no** | **Cases** | **N** | **Controls** | **N** |
| --- | --- | --- | --- | --- |
| GSE35846 | Race: African | 37 | Race: Other | 152 |
| GSE35846 | Gender: Female | 124 | Gender: Male | 65 |
| GSE35846 | BMI: $\geq$30 | 109 | BMI: <30 | 80 |
| GSE19301 | Asthma: Severe | 166 | Asthma: Quiet | 394 |
| GSE47655 | Anaphylaxis: True | 6 | Anaphylaxis: False | 5 |
| GSE17755 | Autoimmune disorder*: True | 191 | Autoimmune disorder: Healthy | 53 |
| GSE38485 | Schizophrenia: True | 106 | Schizophrenia: False | 96 |
| GSE52428 | Influenza time course: Baseline | 41 | Influenza time course: within 3.5 days of infection | 39 |
|  | Total | 780 |  | 884 |

#### *RA, polyarticular JIA, systemic-onset JIA, systemic lupus erythematosus

#### Table S3. GEO Datasets used to validate the SeptiCyte^TM^ TRIAGE signature

| **Dataset accession no** | **Study population** | **Cases** | **N** | **Controls** | **N** |
| --- | --- | --- | --- | --- | --- |
| GSE28750 | Adult | Bacterial sepsis | 10 | Non-infection SIRS | 11 |
| GSE6535 | Adult | Bacterial sepsis | 55 | Uninfected ICU controls | 17 |
| GSE9960 (A) | Adult | Mixed infection bacterial sepsis | 10 | Uninfected ICU controls | 16 |
| GSE9960 (B) | Adult | Gram positive bacterial sepsis | 17 | Uninfected ICU controls | 16 |
| GSE9960 (C) | Adult | Gram negative bacterial sepsis | 18 | Uninfected ICU controls | 16 |
| GSE70311 | Adult | Bacterial sepsis | 5 | Non-infection SIRS | 5 |
| EMTAB4785 | Pediatric | Bacterial sepsis | 17 | Uninfected hospitalised control | 19 |
| GSE11908 | Adult+Pediatric | Bacterial sepsis | 25 | Systemic lupus erythematosis | 54 |
| GSE19491 | Adult+Pediatric | Bacterial sepsis | 52 | Non-infection SIRS | 141 |
| GSE63990 | Adult+Pediatric | Bacterial sepsis | 73 | Non-infection SIRS | 90 |
| GSE13015 | Adult | Bacterial sepsis | 15 | Healthy | 10 |
| GSE28750 | Adult | Bacterial sepsis | 10 | Healthy | 20 |
| GSE60244 | Adult | Bacterial sepsis | 22 | Healthy | 40 |
| GSE69528 | Adult | Bacterial sepsis | 83 | Healthy | 55 |
| EMEXP3567 | Pediatric | Bacterial sepsis | 6 | Healthy | 3 |
| GSE19491 | Adult+Pediatric | Bacterial sepsis | 52 | Healthy | 99 |
| GSE22098 | Adult+Pediatric | Bacterial sepsis | 52 | Healthy | 81 |
| GSE42026 | Pediatric | Bacterial sepsis | 18 | Healthy | 33 |
| GSE64456 | Pediatric | Bacterial sepsis | 89 | Healthy | 19 |
| GSE103119 | Pediatric | Bacterial infection | 35 | Healthy | 20 |
| GSE73464 (A) | Pediatric | Bacterial infection | 52 | Healthy | 55 |
| GSE73464 (B) | Pediatric | Bacterial infection | 23 | Healthy | 16 |
|  |  | Total | 739 |  | 836 |

#### Table S4. Apriori defined list of risk factors for infection

| autoimmune rheumatological disease |
| --- |
| chronic heart disease |
| chronic kidney disease |
| chronic liver disease |
| chronic lung disease |
| chronic neurological disability |
| diabetes |
| inflammatory bowel disease |
| haematological malignancy |
| solid organ malignancy |
| HIV |
| bone marrow transplantation |
| solid organ transplantation |
| immunosuppresion with corticosteroids |
| immunosuppresion (other) |
| pregnancy |
| alcohol misuse |
| Intravenous drug user |
| occupational water exposure |
| occupational animal exposure |
| infectious disease contact |
| developing world travel history within 3 months |
| previous resistant bacterial isolate |
| orthopaedic prosthesis |
| indwelling urinary catheter |
| indwelling intravascular catheter |
| prosthetic heart valves |
| intravascular prosthesis or stent |
| surgery within 3 months |
| hospital admission within 1 year |

####

#### Table S5. GEO Datasets used to validate the combined SeptiCyte^TM^ signature

| **Dataset** | **Population** | **Bacterial cases** | **N** | **Viral cases** | **N** |
| --- | --- | --- | --- | --- | --- |
| GSE60244 | Adult | Bacterial LRTI | 22 | Viral LRTI | 71 |
| GSE73464_GPL6947 | Pediatric | Definite bacterial | 23 | Definite viral | 28 |
| GSE73464_GPL10558 | Pediatric | Definite bacterial | 52 | Definite viral | 94 |
| GSE64456 | Pediatric | Bacterial | 89 | Viral | 111 |
| GSE63990 | Adult | Bacterial | 70 | Viral | 115 |
| GSE40396 | Pediatric | Bacterial | 8 | Viral | 35 |
| GSE6269_GPL96 | Pediatric | *S. aureus, S. pneumoniae* | 73 | Influenza | 18 |
| GSE6269_GPL570 | Pediatric | *S. aureus, S. pneumoniae* | 12 | Influenza | 10 |
| GSE6269_GPL2507 | Pediatric | *S. aureus, S. pneumoniae* | 16 | Influenza | 8 |
| GSE42026 | Pediatric | Bacterial sepsis | 18 | Viral sepsis | 41 |
| GSE69529 | Pediatric | *E.coli, Shigella* Sp. | 121 | Rotavirus | 53 |
|  |  | Total | 504 |  | 584 |
